# Clinical Communication in Rehabilitation

**DOI:** 10.3390/ijerph19127268

**Published:** 2022-06-14

**Authors:** Irene P. Carvalho, Artemisa R. Dores

**Affiliations:** 1 Medical Psychology Unit, Clinical Neurosciences and Mental Health Department, Faculty of Medicine, University of Porto, 4200-319 Porto, Portugal; 2 CINTESIS@RISE, Faculty of Medicine, University of Porto, 4200-450 Porto, Portugal; 3 Center for Rehabilitation Research, School of Health, Polytechnic Institute of Porto, 4200-465 Porto, Portugal; artemisarocha@gmail.com; 4 Laboratory of Neuropsychophysiology, Faculty of Psychology and Education Sciences, University of Porto, 4099-002 Porto, Portugal

Rehabilitation can be a challenging process for both patients and health care professionals. It is associated with a potential loss of function and competence and might represent the possibility of improvement. Thus, frustration, hope, and other complex and mixed reactions are often at work in this process, along with complications pertaining to the actual disease presentation.

Beyond the clinical setting, participants’ daily lives, including family members and daily occupations, can contribute to the quality of rehabilitation and influence the patient’s participation in this process and associated outcomes. The growing recognition of the importance of psychological and social factors, in addition to physical factors, in health approaches has helped to overcome the limitations of the traditional biomedical model in which many professionals have received their training and still practice their professional activity [1,2,3]. A lesion or illness can alter people’s lives in drastic ways as a result of physical, behavioral, cognitive, emotional, or psychosocial changes. Beliefs, expectations, and emotional reactions associated with rehabilitation constitute an individual process that can be as diverse as the variety of biological organs or regions that were affected, functions that became compromised or lost, possibilities of healing, pre-morbid functioning, personality characteristics, previous experiences, and available social support. From difficulty in accepting the problem, or in recognizing its presence, to positive adaptation to the rehabilitation process, multiple reactions might emerge throughout. The way in which healthcare professionals communicate and relate with the patient in the clinical encounter can play a central role in this process, including in the quality of patient participation in rehabilitation and associated outcomes.

Today, communication is recognized as one of the core ingredients of clinical interventions, influencing the development of a patient–health professional relationship that is desirably therapeutic in nature. However, the growing importance attributed to the patient’s active role in health processes, provision of information, communication and emotional support has led to the simultaneous recognition of the necessity of training and repetition for professionals to be able to carry out these principles effectively in their clinical practice [4]. Educational curricula in different health professions have started to include the development of these relational skills, and the effects of such programs have been reported in the literature [5,6].

Research accumulating over the years shows the benefits of competent communication regarding patient adherence to treatment, well-being, clinical biomarkers, and costs for healthcare systems [7,8,9,10]. Conversely, studies have shown that many patients are dissatisfied with the quality of communication with health professionals, which constitutes one of the main reasons for their complaints [11]. Much of this research has been conducted in the fields of medicine or nursing, although much more research is needed in other health areas as well.

Rehabilitation can be experienced with ambivalence, and patient participation and adherence can become a challenge, especially if the program is complex, long, or interferes with patients’ daily routines, the clinical atmosphere generates concerns or is unfriendly, and the benefits (or the underlying condition) are imperceptible or slow to emerge [12,13]. Sensitivity to each situation, empathy, and emotional support can play an important role in the patient’s participation in rehabilitation and associated health outcomes, possibly influencing the quality and quantity of the information that is collected and delivered. Some means of conveying information (e.g., talk, written material, videos) and some models of communicating might be particularly efficacious when compared with others. Complex situations such as the delivery of bad news and strong emotions might emerge, family members might be involved in the interaction, and the rehabilitation plan might extend outside the clinical setting, requiring exercises, lifestyle changes, or other therapeutic modalities at home or autonomously, all of which pose challenges for the success of the therapeutic process.

Evidence regarding communication in rehabilitation is necessary to address the particular challenges that are involved in this vast domain of healthcare that spans from physiotherapy to speech therapy and include areas involving complementary exams such as radiology or cardio-pulmonology, cognitive rehabilitation, medicine, nursing, etc. Papers addressing challenges and communication aspects in the field of rehabilitation are invited for this Special Issue, in particular those combining high academic standards with a practical focus that can illuminate clinical encounters. A breadth of research methods addressing such communication aspects as empathy, relation-, patient- and professional-centered approaches, informing and planning, non-verbal aspects and features of the physical context that facilitate clinical communication, difficult situations and emotions, or interprofessional interactions, with patients of different ages and/or their caretakers, are welcome.
